# Management and Outcomes of Firework-Related Hand Blast Injuries: A Case Series on Surgical Decision-Making, Functional Preservation, and Psychosocial Recovery

**DOI:** 10.7759/cureus.108588

**Published:** 2026-05-10

**Authors:** Abdallah S Samarah, Christian Delcorro, Youssef El Hamshary, Reagan Smith, Gary Schwartz

**Affiliations:** 1 Orthopedic Surgery, Nova Southeastern University Dr. Kiran C. Patel College of Allopathic Medicine, Fort Lauderdale, USA; 2 Occupational Therapy, Nova Southeastern University Dr. Kiran C. Patel College of Osteopathic Medicine, Fort Lauderdale, USA

**Keywords:** blast injury hand, functional recovery, psychosocial impact, rehabilitation process, trauma management

## Abstract

We examined treatment outcomes of firework-related hand injuries through a case series and literature review by evaluating various surgical results and long-term psychosocial recovery. The objective of this study was to evaluate common injury patterns, surgical decision-making, restoration of hand function, and the multidisciplinary approach to physical and psychological recovery following firework-related hand injuries. This study employed a mixed descriptive design, integrating six clinical case reports with a targeted literature review. All six cases were analyzed collectively; however, three representative cases were selected for inclusion in the final manuscript to avoid redundancy while demonstrating the common patterns and treatment approaches associated with blast injuries to the hand. This approach allowed for the presentation of firsthand clinical experiences while contextualizing them within the broader body of published evidence on surgical decision-making for restoring hand function and psychological recovery following firework-related hand injuries, as well as proposing a decision-making framework and providing an educational narrative on multidisciplinary care. The reviewed cases demonstrated recurrent patterns of soft tissue destruction, fractures, tendon injuries, neurovascular compromise, and digit amputations requiring individualized surgical management strategies. The findings additionally emphasized the importance of prioritizing functional preservation when considering digit salvage, as well as the role of multidisciplinary rehabilitation, including occupational therapy and psychosocial support, in optimizing long-term functional and psychological recovery following firework-related hand injuries.

## Introduction

Blast injuries are often severe and present with variable and complex hand injury patterns. Soft tissue, muscle, nerves, vasculature, and bone may be involved, most commonly due to fireworks explosions in civilian settings. Knowledge of the injury pattern and mechanism is essential to guide surgical repair, as presentation varies significantly. While there are numerous proposed treatment algorithms [1,2], there remains limited literature integrating surgical decision-making with long-term functional and psychosocial recovery following firework-related hand blast injuries. Furthermore, algorithms ought not be centered on immediate surgical treatment only, but rather include long-term considerations involving physical, occupational, and psychosocial rehabilitation as a lifelong process to reduce pain, improve range of motion (ROM), and strengthen both physical and psychological abilities to resume daily life [3]. Therefore, this study aims to evaluate common injury patterns, surgical management strategies, and the role of multidisciplinary rehabilitation through representative cases of firework-related hand blast injuries.

Epidemiology and mechanism of blast injuries

Injuries sustained from explosions can be highly variable. The type of explosion (mechanical or chemical), proximity of the victim, material involved in the explosion, and body region affected are all factors that influence the type and severity of injury. The extent of injuries can be attributed to the large amount of energy released within a short period of time [4]. Blast injuries can be categorized into four mechanisms: primary, secondary, tertiary, and quaternary.

Primary blast injuries are caused by the blast wave itself as it moves through the body, leading to internal injuries that may not be readily apparent. Secondary blast injuries are caused by debris displaced by the blast wave, and they account for most injuries, including penetrating wounds. If the blast wind is strong enough, the victim may be displaced, leading to impact with another object, which accounts for tertiary injuries. Quaternary blast injuries include any injury not included in previous categories.

While most blast injuries occur in military settings, civilian cases are often related to accidental firework injuries or illegal explosions. The number of firework-related blast injuries has been steadily increasing every year in the United States, with injuries typically occurring in July, with smaller peaks in June and January, affecting mostly male pediatric and young adult groups [5]. These injuries are commonly associated with accidental discharge of fireworks while victims are nearby or holding them. Prior studies have demonstrated that injuries to the hands and eyes are most common, with a marked decrease in hand function and blindness as outcomes following severe injuries [5].

Injury patterns

Injury patterns can be highly variable depending on the mechanism of injury. Primary blast injuries occur due to the blast wave, leading to extensive barotrauma and damage to air-filled organs, such as the tympanic membrane and middle ear, lungs, brain parenchyma, eyes, and abdomen [4]. These injuries may not be readily apparent on presentation and may have a delayed onset; therefore, evaluation is warranted in patients presenting with symptoms related to these organs.

Secondary injuries are caused by displaced debris and can result in significant penetrating wounds, amputations, bone fractures, skin lacerations, joint dislocations, neurovascular injuries, and soft tissue injuries [6]. The severity of tertiary injuries is dependent on the intensity of the explosion and the type of object with which the patient impacts. Injuries sustained can be similar to secondary injuries, with the addition of compartment syndrome and crush injuries. Quaternary injuries include any other injuries sustained from an explosion not included in previous categories, including burns, crush injuries, and inhalational injuries [7].

Initial assessment and management

Because firework-related hand blast injuries may differ due to varying detonation mechanisms, initial medical management by emergency medical services at the scene is imperative. This includes a primary survey as outlined by the Advanced Trauma Life Support (ATLS) guidelines taught by the American College of Surgeons. Assessment begins with securing the airway, ensuring adequate breathing, managing circulation and hemorrhage control, evaluating disability (neurological status), and full exposure of the patient to identify any hidden injuries before covering to prevent hypothermia [7,8].

Following ATLS protocols has been shown to significantly reduce preventable deaths following trauma from 30% (n = 100) to 15% (n = 100) and potentially preventable deaths from 40% (n = 100) to 25% (n = 100) [9].

Diagnostic workup

Upon arrival at the hospital, the secondary survey determines the extent of injuries. Administration of vaccines, such as tetanus, and antibiotics should be addressed. While all patients should be evaluated for pulmonary, gastrointestinal, neurologic, cardiovascular, facial, and auditory injuries as described in the “Injury patterns” section, this study focuses on musculoskeletal injuries affecting the hands.

After bleeding control, X-rays are the primary imaging modality to assess damage beyond soft tissue injury. A review shows a trend in finger involvement moving from the radial to the ulnar side, with the thumb and first web space being most affected by such injuries [2].

Surgical management

The consensus in the surgical treatment of firework-related hand blast injuries is debridement of bone and soft tissue as a first step to remove dead, damaged, or infected tissue, thereby preventing further complications. Following this, bone and tendon injuries are addressed using various techniques depending on the site of injury [10].

When tissue and limb preservation is severely compromised with low chances of success, functional preservation is prioritized in the hand, particularly involving the thumb, as its function contributes substantially to overall hand function. This explains reconstructive guidelines that emphasize restoration of sensation and opposition as primary goals in first web space and thumb surgeries [11].

Long-term complications

Long-term psychosocial consequences can contribute to the development or exacerbation of conditions such as anxiety, depression, altered body image, and symptoms of post-traumatic stress disorder (PTSD). Patients may not meet full diagnostic criteria for PTSD but may exhibit subthreshold symptoms, including hyperarousal, nightmares, avoidance, flashbacks, and sleep disturbances. These symptoms can create difficulties in daily life and delay rehabilitation and recovery.

Furthermore, psychosocial stress is closely linked to physical recovery and pain perception. Patients experiencing severe psychosocial distress are less likely to adhere to therapy and follow-up treatment, decreasing the likelihood of functional recovery. Early psychological support, including therapy and coping strategies, is therefore essential for positive functional outcomes and overall recovery.

Patients who undergo amputation or severe hand and digit injury may also struggle with daily activities and maintaining employment. Adjustment after loss of hand function is often difficult, highlighting the importance of occupational therapy.

Psychosocial concerns

Although there is limited literature addressing the direct psychosocial impact of sustaining a firework injury to the hand, there is substantial research discussing the psychosocial consequences of the broader category of traumatic hand injuries. Individuals who sustain traumatic hand injuries may suffer long-term psychological impacts, including PTSD, work avoidance, appearance and body image issues, sleep disturbance, and pain [12]. A cross-sectional study by Agarwal et al. in 2020 found that, with only 26 (13%) of the participants qualifying clinically for a PTSD diagnosis, 174 (87%) of the participants experienced subthreshold PTSD symptoms after experiencing a traumatic injury (n = 200) [13]. This emphasizes the need for greater attention to be placed on addressing subthreshold PTSD symptoms, rather than addressing only those who are clinically diagnosed with PTSD. Aside from experiencing PTSD, a study by Gustafsson et al. in 2003 found that approximately one-third of the participants who had experienced a traumatic hand injury displayed signs of a mood disorder (depression, anxiety, or both) after their injury [14]. Other psychosocial impacts that are often experienced after sustaining a traumatic hand injury include flashbacks, nightmares, concentration difficulties, intense emotions (disgust, irritability, and hostility), increased startle reactions, phantom sensations, sexual dysfunction, avoidance, denial, gaze aversion, alcohol/drug abuse, role change, and marital distress [12,15]. Role change can be a particularly impactful psychosocial component. The individual’s physical difference due to the traumatic hand injury will then affect how they participate in their daily roles (student, mother, spouse, dog owner, employee, etc.), resulting in feelings of dissatisfaction and distress [15].

Although occupational therapists (OTs) working as hand therapists are uniquely positioned to address the physical and psychosocial impacts of traumatic hand injuries, a quantitative survey design study by Chown et al. in 2018 found that the majority of hand therapists practice using a biomechanical approach, addressing only the physical components [12]. However, a systematic review by Roll and Hardison in 2017 emphasized the importance of utilizing a biopsychosocial approach for reducing patient pain and optimizing functional outcomes when compared with a model that does not address the psychosocial component of these injuries [16]. This finding further emphasizes the impact of psychosocial factors on physical recovery, reinforcing the need for hand therapists to more adequately address the psychosocial component during treatment. When addressing the psychosocial component of traumatic hand injuries, hand therapists should assess the coping strategies clients use to manage psychosocial distress and evaluate how these approaches influence their recovery and overall adjustment [15]. To target the psychosocial aspects of traumatic hand injuries, hand therapists may incorporate interventions such as massage, guided imagery, meditation, relaxation techniques, graded motor imagery, education on pain management and adaptive coping strategies, and assistance in identifying supportive resources [12,15,17]. The implementation of these psychosocial interventions should receive greater emphasis from hand therapists to increase functional outcomes and psychological well-being in those who have experienced traumatic hand injuries, such as firework-related injuries.

In this study, we present three representative cases of firework-related blast injuries and discuss the implications of the performed procedures within the broader context of established treatment strategies. The aim of this study is to evaluate common injury patterns, surgical decision-making, and long-term functional recovery following firework-related hand injuries, while emphasizing the importance of multidisciplinary rehabilitation, including psychosocial support and occupational therapy, in optimizing both physical and psychological outcomes.

## Materials and methods

Study design

This study employed a mixed descriptive design, integrating six clinical case reports with a targeted literature review. This approach allowed for the presentation of firsthand clinical experiences while contextualizing them within the broader body of published evidence on surgical decision-making to restore hand function and psychological recovery following firework-related hand injuries.

Study participation

Six clinical cases involving firework-related hand injuries were retrospectively reviewed. Cases were included based on the presence of firework-related hand blast trauma requiring surgical evaluation and management. All six cases were analyzed to identify mechanisms of injury, injuries sustained, psychosocial issues, treatments, and operative interventions performed to repair the damage caused by fireworks. Three representative cases demonstrating common injury patterns and variations in surgical management were selected for detailed presentation in this review to avoid redundancy while highlighting key clinical and rehabilitative considerations. Patient information was de-identified to protect confidentiality.

Literature search

A targeted literature search was conducted to identify studies addressing the management, surgical outcomes, rehabilitation, and psychosocial implications of firework-related hand injuries and traumatic hand blast injuries. Psychological factors, including barriers to recovery and long-term psychosocial outcomes, were also reviewed to better contextualize multidisciplinary recovery approaches.

## Results

Demographics

We identified six cases between 2013 and 2019 of patients who underwent surgical treatment of their hands for firework-related blast injuries. All patients included in the review were men (n = 6; 100%). Ages ranged from 17 to 51 years, with a mean age of 31.5 years and a median of 27.5 years. In accordance with national trends, the most common month for injury was July, accounting for four of the six cases [5] (66.67%), followed by December, with one of the six cases (16.67%), and October, also with one of the six cases (16.67%). It is noteworthy that all July admissions occurred exclusively on July 4th. There were four right-handed patients (66.7%), one left-handed patient (16.7%), and one patient with unknown hand dominance.

Injury patterns

All reviewed cases suffered severe soft tissue or degloving injuries. Fifty percent of the cases had bilateral hand injuries, while the remaining cases involved the dominant hand only. Following soft tissue injuries, digital and hand amputations had the highest prevalence at 83.3% of cases, followed by proximal phalanx and metacarpal fractures, which were both seen in 66.7% of cases.

Additionally, patients suffered various non-hand-related injuries, which are detailed below in Table 1, showing the complexity associated with firework blast injuries.

**Table 1:** Detailed description of types of non-hand injuries encountered in the case series (n = 6)

System	Specific injury	Patients (n/6)
Dermatologic/musculoskeletal (non-hand)	Bilateral knee abrasions	1 (16.67%)
	Abdominal abrasions and burns	1 (16.67%)
	Torso blast wounds	1 (16.67%)
Eyes (ocular trauma)	Bilateral corneal abrasions and ulcerations	1 (16.67%)
Head and neck	Penetrating Zone I neck wound with platysma violation	1 (16.67%)
Abdomen (external + internal trauma)	External abdominal blast injury	1 (16.67%)
	Internal abdominal blast injuries	1 (16.67%)
Groin/genitourinary	Right groin blast injury	1 (16.67%)
	Right testicular avulsion	1 (16.67%)
Lower extremity	Right thigh blast injury	1 (16.67%)
Respiratory/airway	Suspected inhalation injury	1 (16.67%)
Systemic/physiologic	Profound hypotension/shock at arrival	1 (16.67%)
None (no non-hand injuries)	No extracarpal traumatic injuries at presentation	2 (33.33%)

Treatment and complications

All patients in the reviewed cases underwent surgical debridement and fracture fixation. K-wires were used extensively for fixation of fractures in all patients. Further treatments included muscle and tendon repairs, amputations, and soft tissue repair using flaps. Lastly, it is worth noting that psychiatric care was established and addressed in only 2/6 patients.

Blast injury of the hand - case reports

Case 1

**Figure 1:**
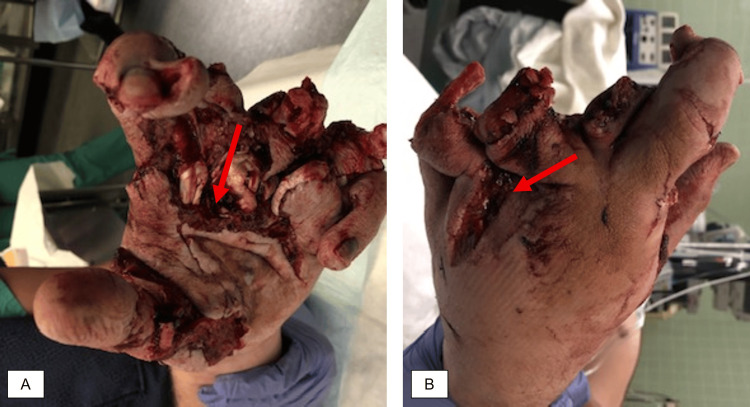
Preoperative clinical findings The injury photos demonstrate extensive soft tissue loss and multiple open injuries and lacerations to the volar aspect (A) and dorsal aspect (B) of the left hand (red arrows).

A 25-year-old left-handed male who works as an aviation mechanic presented with a blast and degloving injury to his left hand following an explosion while handling Fourth of July firecrackers. The injury resulted in extensive soft tissue loss, multiple open comminuted fractures, and a flexor digitorum superficialis tendon laceration of the index finger (Figure 1).

**Figure 2:**
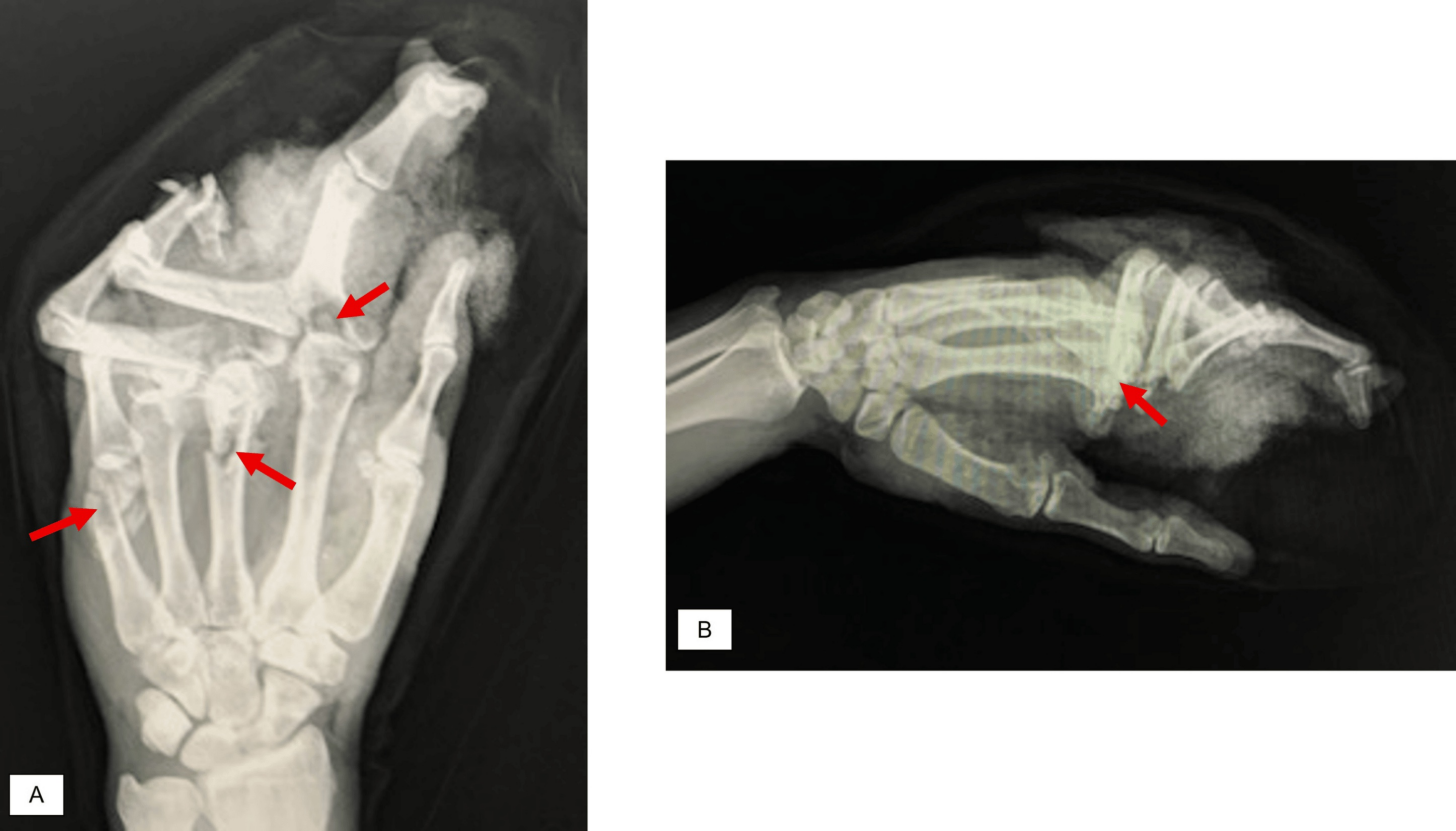
Preoperative radiographs of the left hand The preoperative injury radiographs demonstrate multiple fractures of the metacarpals and proximal phalanges (A and B) (red arrows).

The patient also sustained superficial abrasions on both knees after falling post-blast, with no underlying fractures. Radiographs revealed comminuted fractures involving the second, third, and fifth metacarpals; the proximal phalanges of the second through fourth digits; and the middle phalanges of the second and third digits, with displacement of the third and fourth proximal phalanges (Figure 2).

On admission, the patient received standard prophylactic measures, including tetanus prophylaxis, intravenous ceftriaxone for antibiotic coverage, intravenous morphine for analgesia, and wound irrigation.

**Figure 3:**
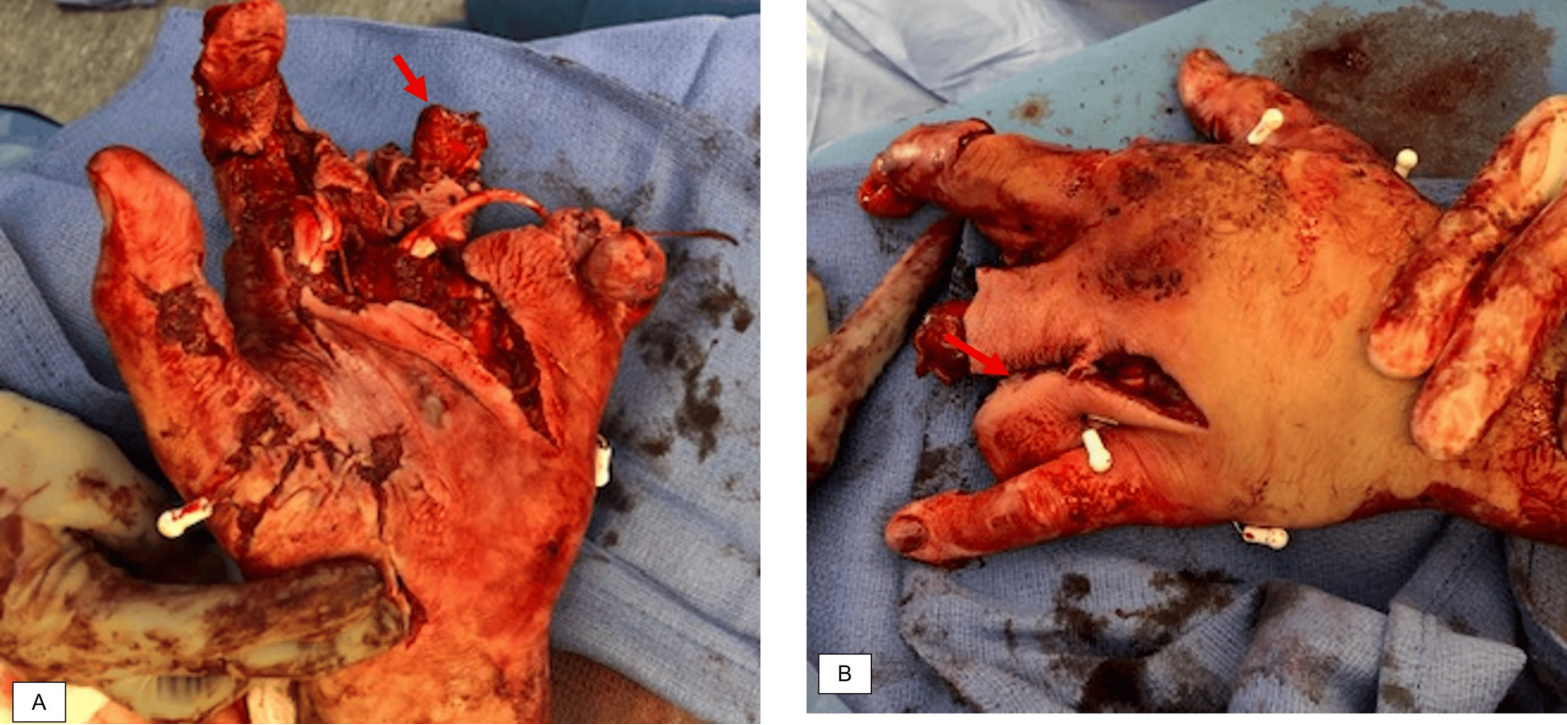
Intraoperative photos of the initial debridement procedure The intraoperative photos of the (A) volar aspect and (B) dorsal aspect of the left hand demonstrate areas of debridement of the intrinsic muscles and amputations of the long and ring fingers (red arrows).

The patient underwent emergent operative management consisting of wound and open fracture debridement, repair of the thenar muscles and dorsal first interosseous muscle, and amputations of the long finger at the distal metacarpal level and the ring finger at the proximal phalanx level (Figure 3).

**Figure 4:**
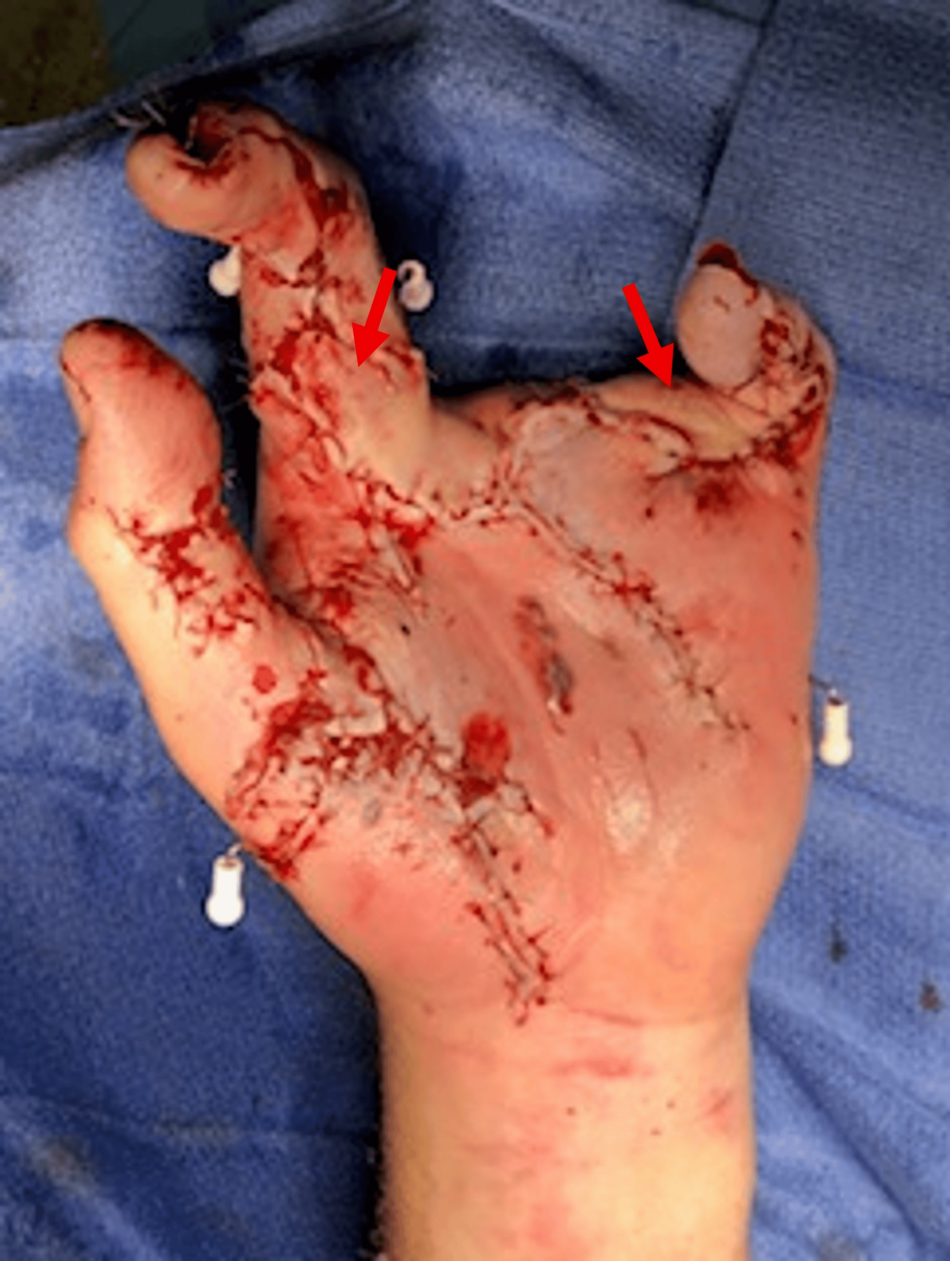
Intraoperative photos demonstrating flap coverage Intraoperative photograph demonstrates the rotational flaps covering the small and index fingers (red arrows).

A spare-part reconstruction technique was used, in which a dorsal pedicle flap from the amputated ring finger was rotated to cover a volar defect on the small finger, and a rotational flap from the amputated long finger was utilized to cover a volar wound on the index finger (Figure 4).

**Figure 5:**
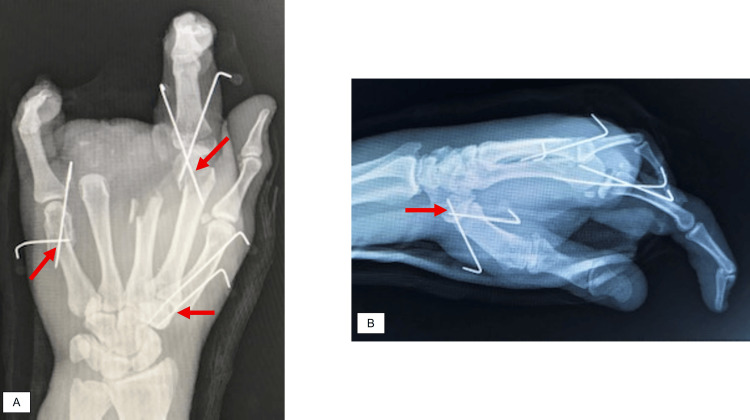
Postoperative radiographs after skeletal fixation An anteroposterior view (A) and lateral view (B) of the left hand demonstrate skeletal fixation of the second and fifth metacarpal fractures as well as the first CMC joint (red arrows). CMC, carpometacarpal

Skeletal stabilization was achieved with pin fixation of the first carpometacarpal (CMC) fracture-dislocation and fractures of the second and fifth metacarpals (Figure 5).

**Figure 6:**
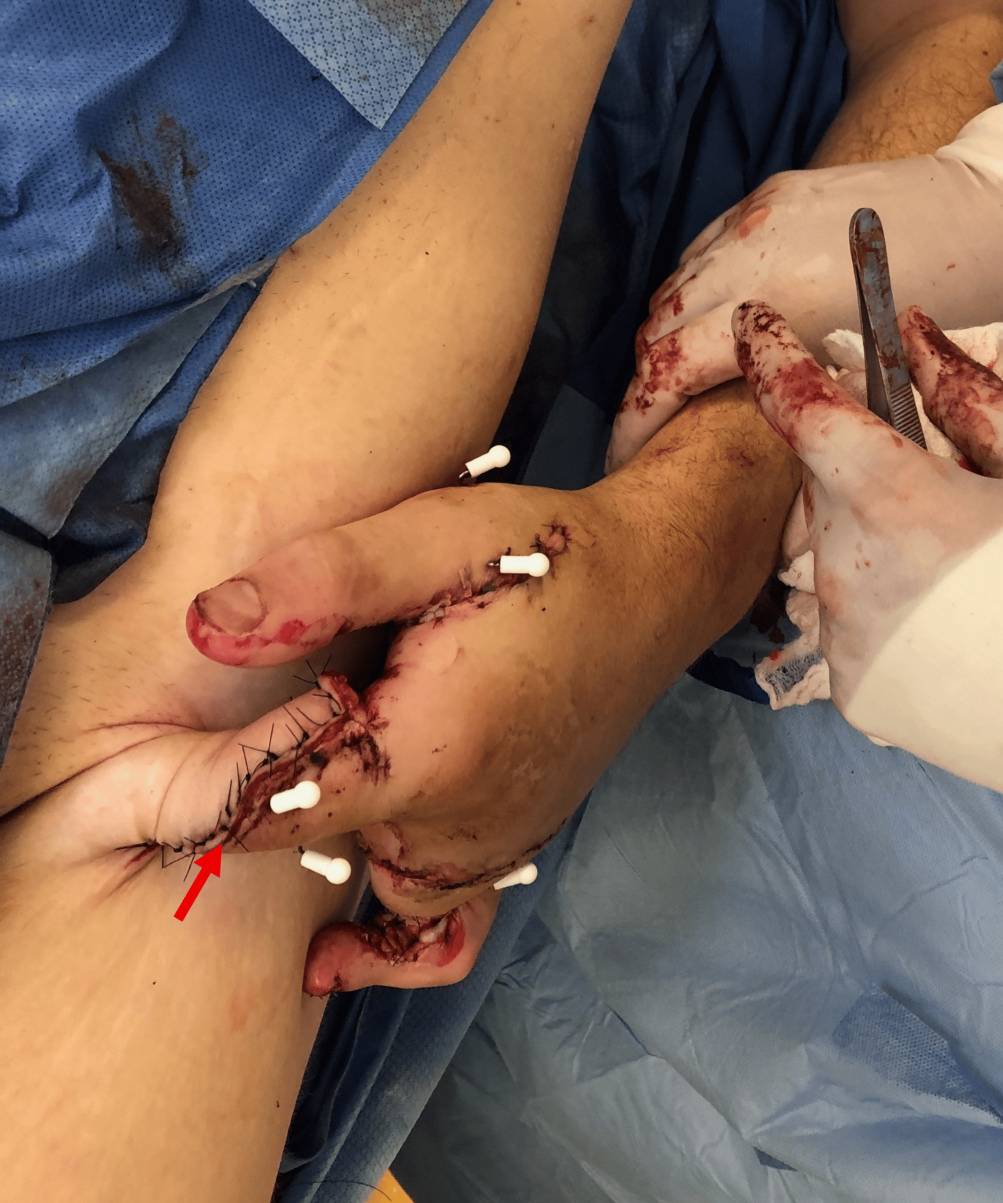
Reconstruction with a pedicled groin flap Intraoperative photograph demonstrating the pedicled groin flap (red arrow).

Twelve days postoperatively, necrosis developed in the volar flap of the index finger. The affected tissue was debrided, and revision amputation with a left groin pedicled tubed flap was performed for coverage (Figure 6).

The patient was subsequently discharged from the hospital. He received outpatient occupational hand therapy for approximately six months to aid functional recovery. The patient reported satisfaction with his healing and functional capability and reported no other symptoms or difficulties. A summary of the progression of treatment and recovery is presented in Table 2.

**Table 2:** Case 1: summary of events

Timepoint	Event
Day 0	Blast injury to the left hand with extensive soft tissue damage and multiple fractures. Patient taken to the operating room for debridement and amputation of the long and ring fingers; received tetanus prophylaxis and ceftriaxone.
Day 11	Gangrene developed in the index finger
Day 12	Debridement of the index finger and application of a pedicled groin flap
Five weeks post-injury	Occupational hand therapy began to aid functional recovery
Six months post-injury	Patient reported satisfaction with recovery and functional status

Case 2

**Figure 7:**
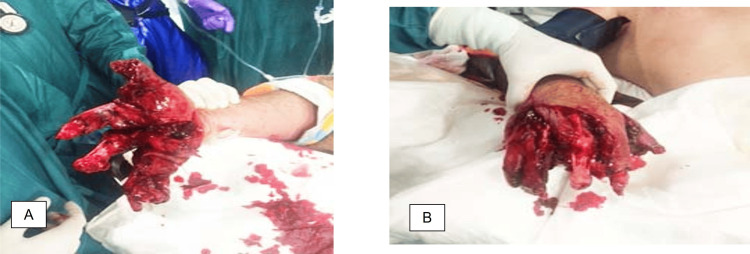
Preoperative images of the right hand Preoperative images of the volar (A) and dorsal (B) aspects of the right hand demonstrating the mangled hand with multiple open wounds and extensive soft tissue injury.

A 45-year-old right-hand-dominant male with a past medical history of diabetes on metformin, hypertension on lisinopril, and depression on Abilify was brought to the hospital following discharge of a firecracker in his right hand. Initial presentation demonstrated a mangled right hand with several open fractures and extensive soft tissue injury over the dorsal and volar aspects of his hand (Figure 7).

**Figure 8:**
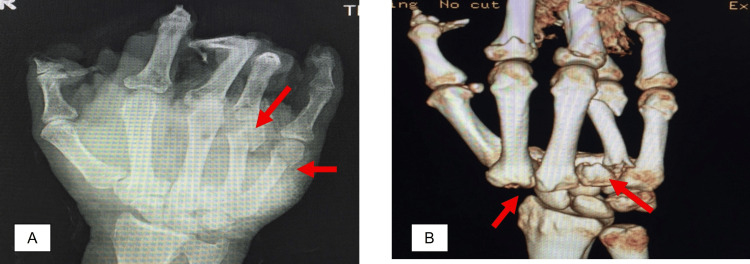
Preoperative radiographs and CT scan Anteroposterior radiographs (A) and a coronal CT scan (B) demonstrating multiple fractures of the metacarpals and phalanges, as well as multiple CMC fracture-dislocations (red arrows). CMC, carpometacarpal

The patient was anxious and agitated on arrival and was unable to tolerate a full evaluation, leading to sedation and intubation. On further evaluation of the right hand, it was noted to be mangled from the proximal metacarpals, with near-complete destruction of the third and fourth fingers, as well as the index finger, with preservation of the proximal phalanx of the thumb and proximal two phalanges of the fifth finger. X-rays demonstrated multiple fractures of the metacarpals and phalanges, as well as multiple CMC fracture-dislocations (Figure 8).

The patient was taken to the operating room by the trauma team, at which time it was noted that the complex wound involved the entire volar surface as well as the dorsal surface of the hand, with the middle and ring fingers essentially skeletonized and devoid of skin. The extensor tendons appeared to be intact in all digits. The remainder of the palmar arch was not easily identified and was severely damaged by the injury. The thumb and fifth digit appeared to have some distal flow. The remaining digits did not have any perfusion. On evaluation, the radial and ulnar pulses were palpable and audible on Doppler examination, with no Doppler-audible pulses of the palmar arches nor of the digits themselves. There was coolness to the index, long, ring, and small fingers, as well as the thumb.

**Figure 9:**
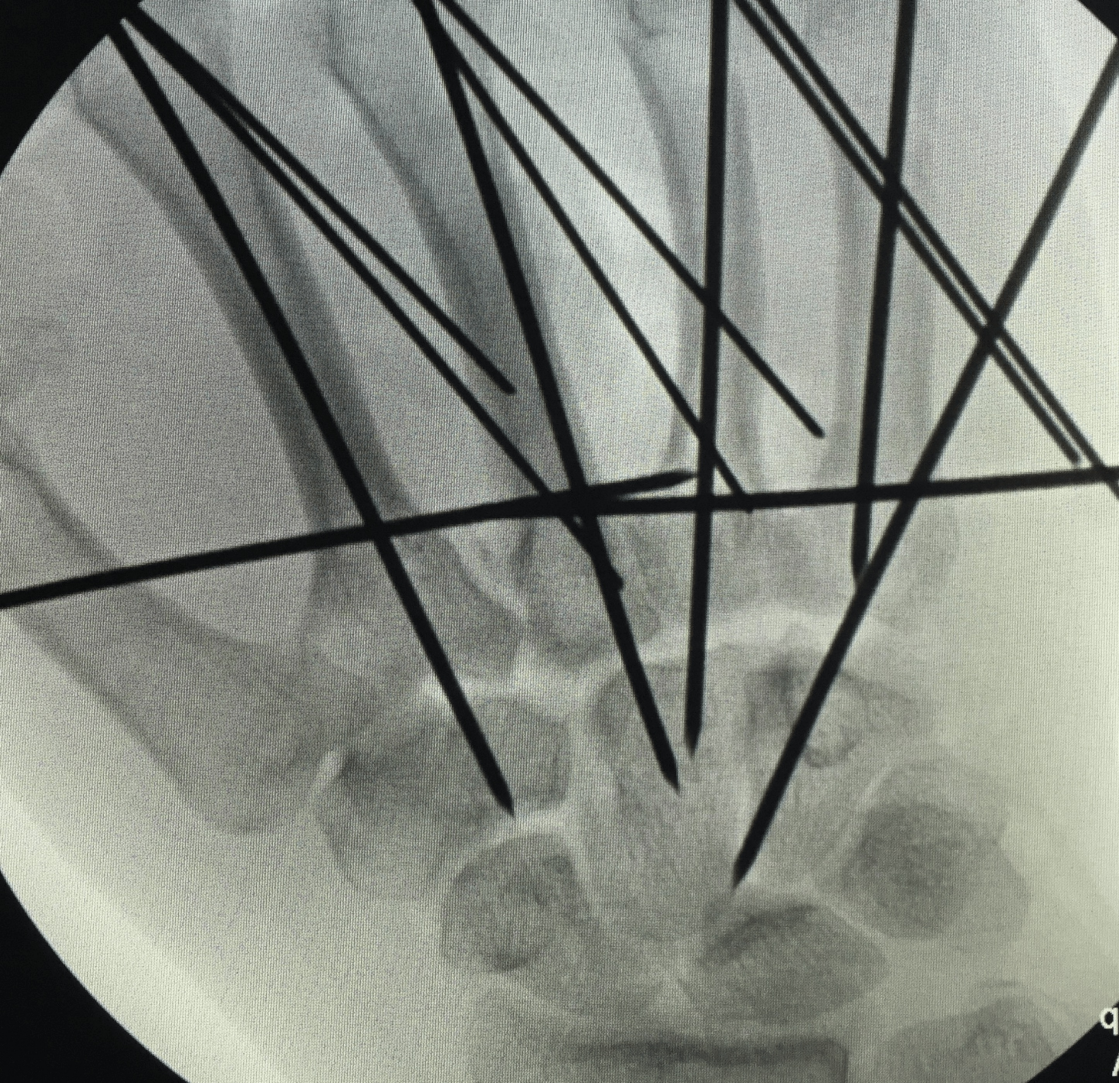
Postoperative radiographs of the right wrist Anteroposterior radiograph of the right wrist demonstrating fixation of the CMC dislocations and metacarpal fractures with multiple Kirschner wires. CMC, carpometacarpal

The patient was taken to the OR and underwent open reduction and internal fixation for the multiple right-hand/wrist injuries two days following the injury. The metacarpal fractures, phalangeal fractures, and CMC dislocations were reduced and stabilized with multiple Kirschner wires (Figure 9).

**Figure 10:**
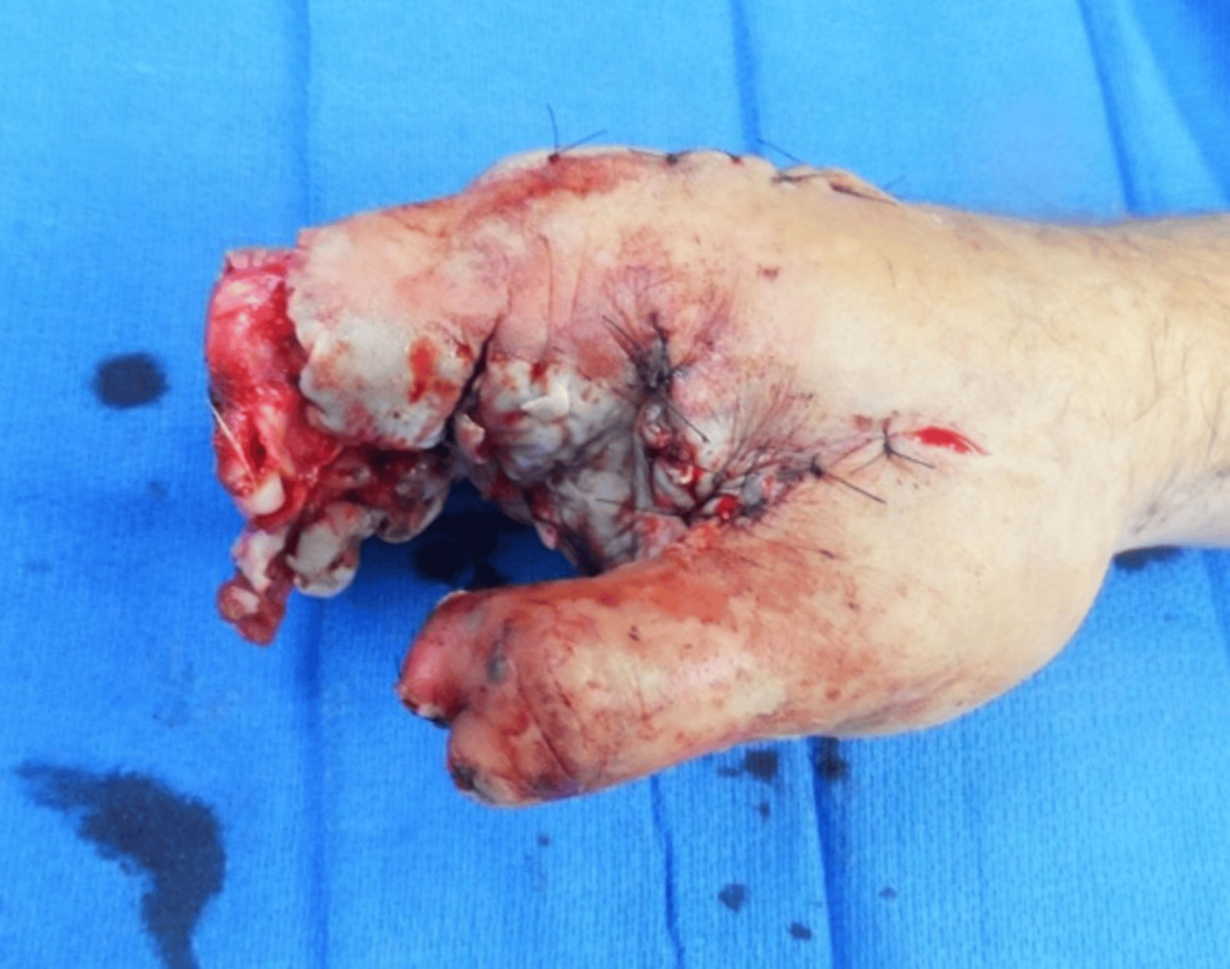
Postoperative photo of the right hand after digital amputation The radial aspect of the hand is seen demonstrating the amputations of the long and ring fingers.

Nine days from presentation, the patient underwent amputation of the right index finger at the metacarpophalangeal joint and the long and ring fingers at the proximal portion of the proximal phalanx (Figure 10).

**Figure 11:**
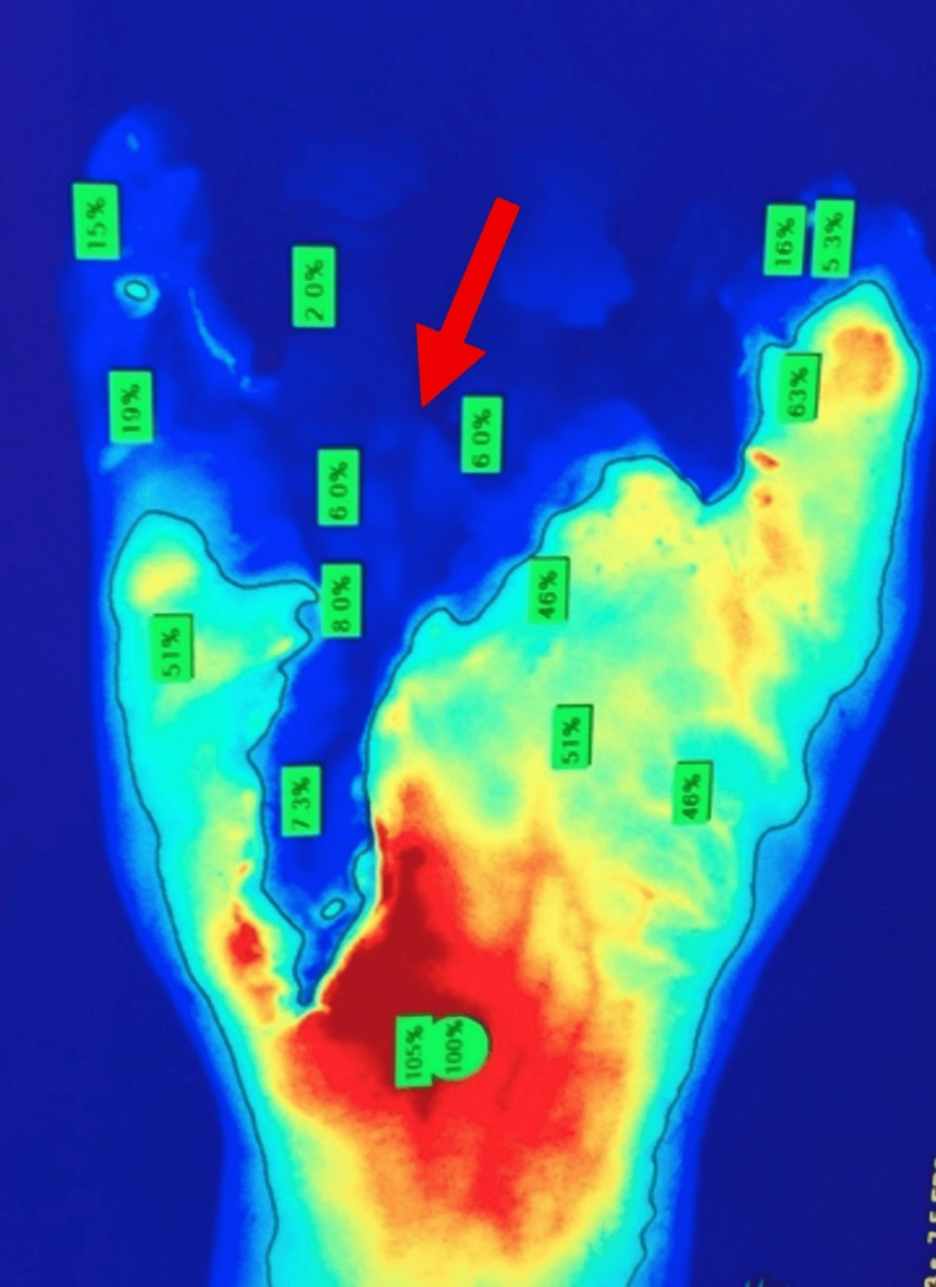
SPY imaging of the right hand Intraoperative SPY/ICG imaging of the right hand demonstrates no blood flow or viable tissue to the right index, long, or ring fingers, as they were completely gangrenous (red arrow). ICG, indocyanine green

This procedure was indicated as no blood flow or viable tissue was seen on SPY perfusion imaging to the right index, long, or ring fingers, and they were completely gangrenous (Figure 11).

**Figure 12:**
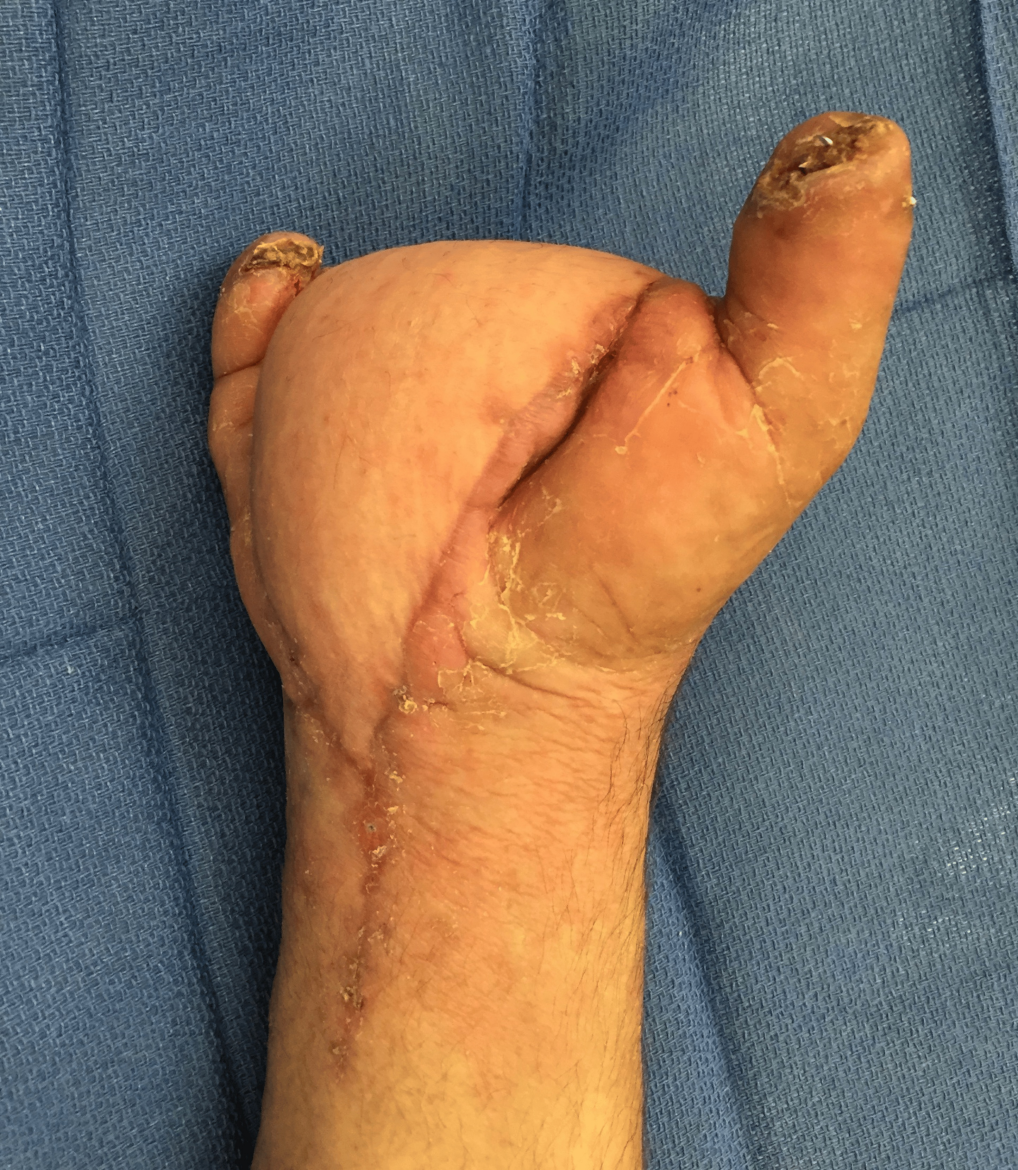
Anterolateral thigh flap Postoperative photograph of the anterolateral thigh free flap, showing preservation of the thumb and a portion of the small finger.

Thirteen days later, the right hand was reconstructed with a right anterolateral thigh free flap (Figure 12).

The patient was discharged in stable condition and followed up with the psychology/neuropsychology service. Following the significant traumatic injury and long hospitalization, he displayed intact cognition and a stable mood with appropriate insight and coping mechanisms. Table 3 summarizes the course of treatment and subsequent recovery.

**Table 3:** Case 2: summary of events

Timepoint	Event
Day 0	Blast injury to the right hand resulting in a mangled hand and several open fractures with extensive soft tissue injury. Patient taken to the operating room for skeletal stabilization.
Day 7	Gangrenous changes seen in the index, long, and ring fingers
Day 9	Amputation of the index, long, and ring fingers
Day 22	Wound coverage with an anterolateral thigh free flap
Five weeks post-injury	Hand therapy began to aid functional recovery
Four months post-injury	Patient followed up with the psychology/neuropsychology service

Case 3

**Figure 13:**
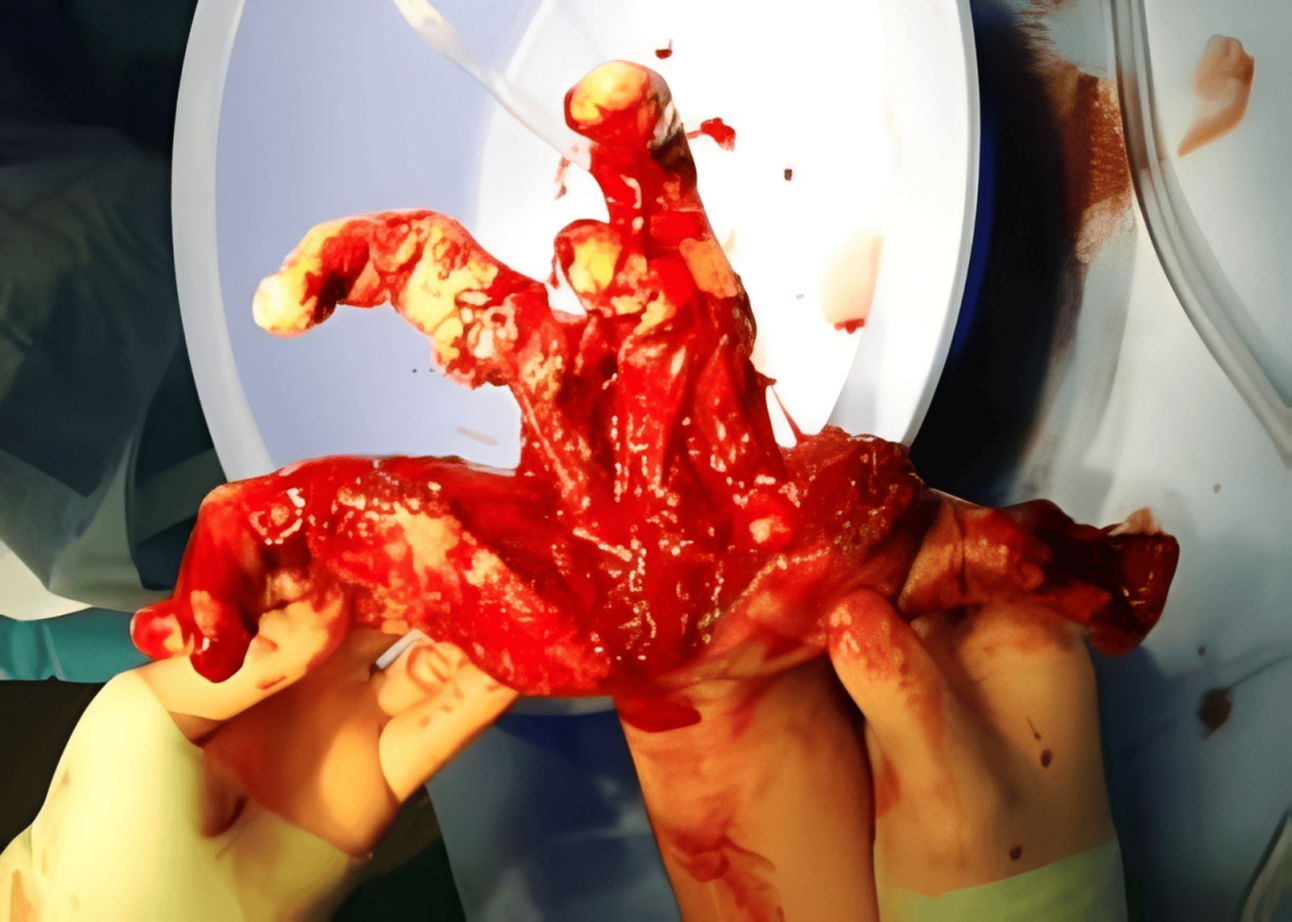
Preoperative photograph of the right hand Preoperative photograph of the right hand demonstrating multiple stellate lacerations with severe soft tissue disruption.

A 21-year-old right-hand-dominant male presented to the emergency room with blast wounds to the right hand and torso from a firework explosion. Upon physical examination, multiple abrasions and burns to the abdomen were noted. The patient was then intubated for possible inhalation injury. The right hand had multiple stellate lacerations associated with severe soft tissue disruption (Figure 13).

**Figure 14:**
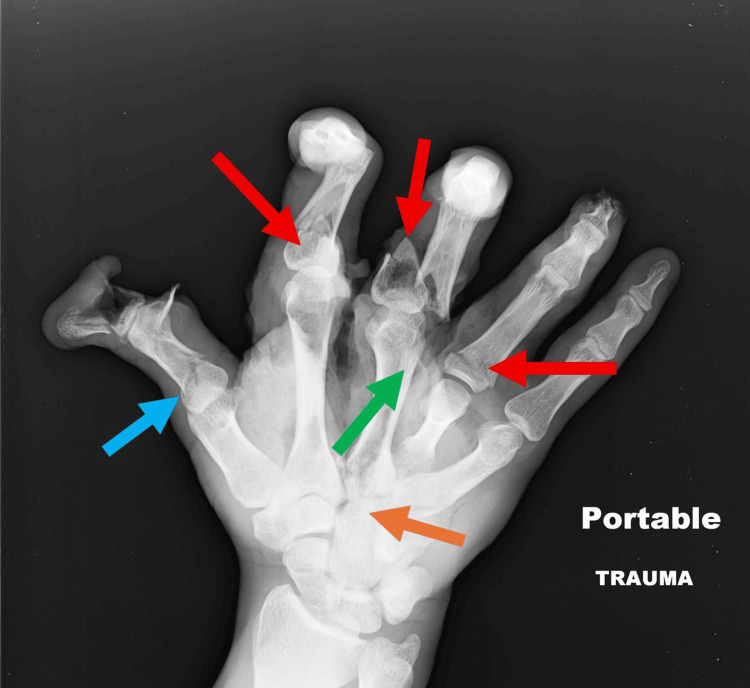
Preoperative radiograph of the right hand Preoperative anteroposterior view of the right hand demonstrating fractures at the base of the proximal phalanx of the index-ring fingers (red arrow), fracture of the proximal and distal phalanx of the thumb (blue arrow), fracture of the third metacarpal (green arrow), and dislocations of the third-fifth CMC joints (orange arrow). CMC, carpometacarpal

The first CMC joint was dislocated on examination. X-rays demonstrated displaced fractures of the base of the proximal phalanges of the index, long, and ring fingers, as well as displaced fractures of the distal and proximal phalanges of the thumb. There was also a fracture of the base and shaft of the third metacarpal with dorsal dislocation of the third-fifth CMC joints. There were fractures of the distal phalanx of the index and long fingers (Figure 14).

**Figure 15:**
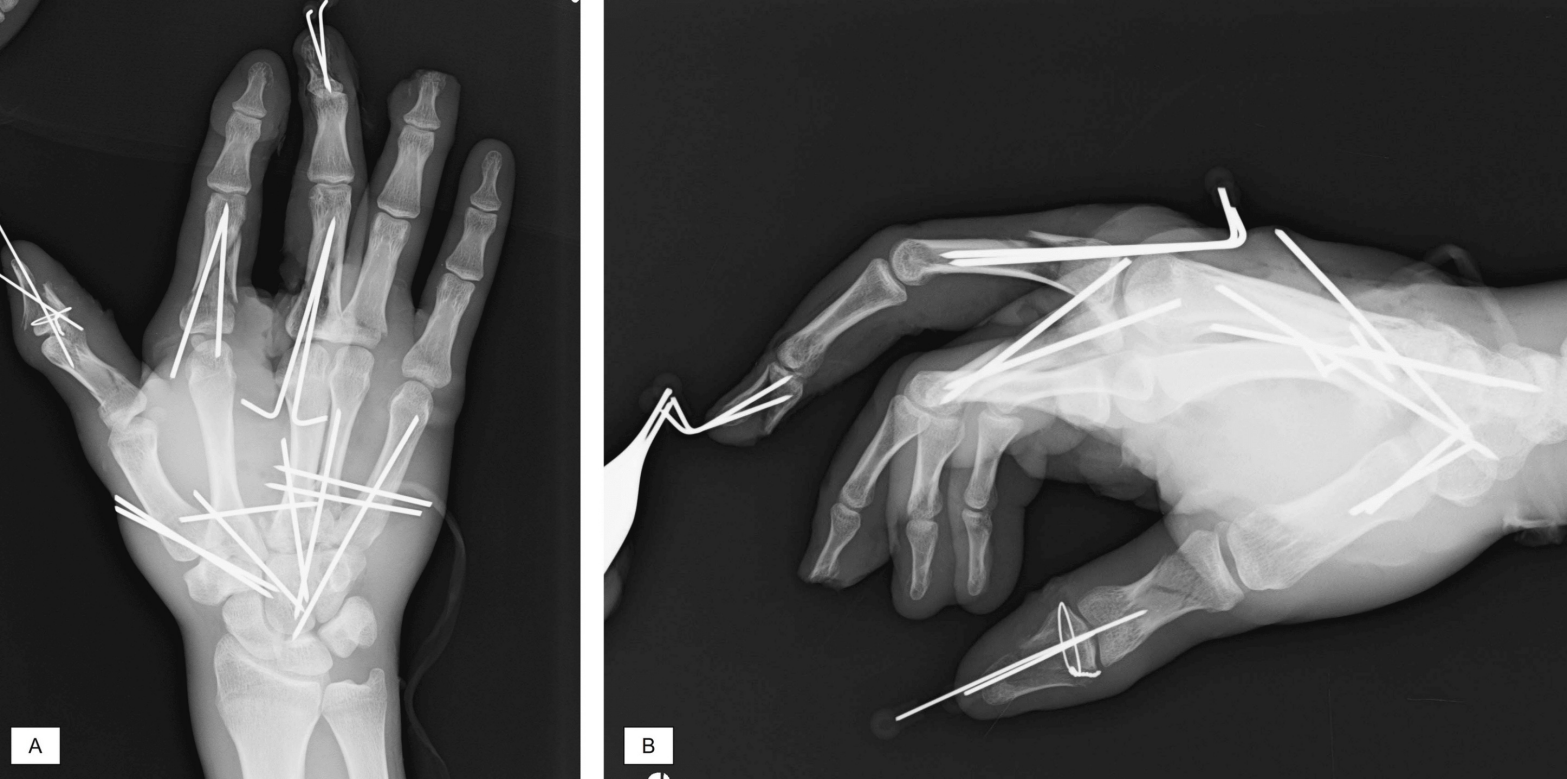
Postoperative radiographs of the right hand Postoperative oblique view (A) and lateral view (B) of the right hand demonstrating stabilization of multiple metacarpal and phalangeal fractures, as well as the third CMC joint.

The patient was then taken to the operating room, at which time most of the fractures were stabilized with multiple Kirschner wires. In subsequent operative procedures, some of the other fractures were stabilized, while the soft tissue was observed for vascularization (Figure 15).

The decision not to immediately amputate the fingers stemmed from the appearance of sufficient vascularization to allow most of the fingers to survive, as well as the possibility of performing a filet flap later.

**Figure 16:**
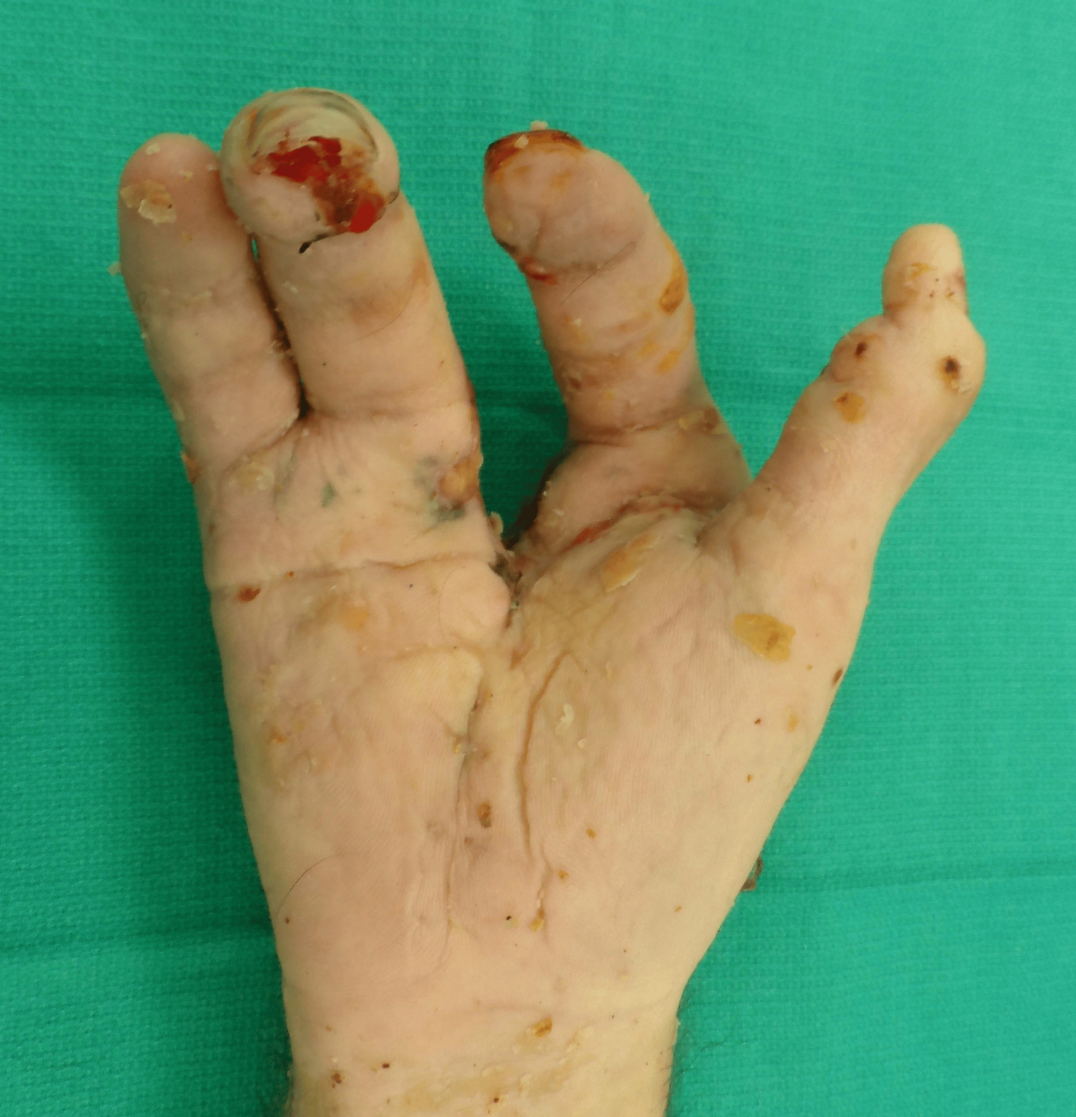
Postoperative image after amputation of the third ray Postoperative photograph demonstrating the volar aspect of the right hand after third metacarpal ray amputation.

The right long finger remained dusky, subsequently became gangrenous, and lacked sensation distal to the proximal phalanx. The long finger was eventually amputated at the CMC joint (Figure 16).

The patient was eventually discharged home and continued with an outpatient occupational therapy program for approximately nine months. He was pleased with his healing. The progression of treatment and recovery is summarized in Table 4.

**Table 4:** Case 3: summary of events CMC, carpometacarpal

Timepoint	Event
Day 0	Firework explosion to the right hand and torso. Patient intubated for possible inhalation injury
Day 1	Debridement of multiple fractures and soft tissue
Day 3	Duskiness of long finger identified
Day 5	Fixation of right second and third CMC dislocations, proximal and distal phalangeal fractures
Day 7	Gangrenous changes identified in the long finger
Day 8	Amputation of long finger
Day 9	Discharge from the hospital
Three weeks post-injury	Occupational hand therapy initiated
Nine months post-injury	Patient reported satisfaction with recovery and functional status

## Discussion

Comparison of cases and literature

Decision-Making

The three cases highlighted in this paper demonstrate the complex process when making decisions for a patient who presents to the emergency room with a blast injury. The first priority should always be the initial stabilization of the patient and a thorough trauma evaluation. As seen in our cases and the reviewed literature, a blast mechanism of injury typically produces occult or remote injuries, making it essential to examine the entire patient and not just the hand [4].

Once the patient is stabilized, the focus should shift to diagnosing and treating the hand injury. This process should also begin with a physical examination aiming to identify the zone of injury, identify open fractures, devitalized tissue, assess vascular status, and evaluate nerve and tendon integrity [18,19]. The physical exam should be supplemented by radiographic imaging, usually starting with a plain film X-ray to identify the extent of skeletal damage, joint dislocations, and retained foreign bodies. In many cases, imaging was able to confirm the degree of comminution and CMC instability. The imaging allowed for preplanning of fixation strategies to repair skeletal fractures (e.g., multiple K-wires and plates in Cases 1, 3, 5, and 6).

A central decision-making point when dealing with severe hand injuries is whether to prioritize digit preservation or overall hand function. Though conserving digits is desirable, aggressive attempts to preserve digits in the setting of hypoperfusion or nonviable tissue can increase infection risk, delay wound healing, prolong hospitalization, and ultimately still result in late amputation. It is essential that the surgeon weigh the probability of meaningful functional recovery of the digit vs possible complications, as well as the impact of multiple staged procedures on a patient’s physical and psychological well-being. This highlights the importance of honest counseling with patients and families regarding the prognosis and the probability of salvage of the digit vs staged amputation.

When making decisions after a blast injury, one should also consider social factors associated with the patient such as age, handedness, occupation, and comorbidities in a psychosocial context. Comorbidities such as diabetes and smoking status should be considered when deciding between attempting digit salvage vs early amputation due to the potential for poor wound healing and the increased risk of developing an infection [13].

It is also important to recognize that these decisions do not occur in isolation; they are best made through discussion with a multidisciplinary team. There should be collaboration between the orthopedic surgeon, trauma surgeon, plastic surgeon, anesthesiologist, intensivist, occupational/physical therapist, and psychological services to best determine immediate priorities as well as long-term functional goals for the patient. Early psychological assessment also has many potential benefits, such as identifying the patient’s risk for anxiety, depression, or PTSD, which can significantly improve a patient’s adherence to rehabilitation and perceived quality of life and outcomes [13].

Decision-making after a blast injury is a complex and dynamic process that begins in the trauma bay with lifesaving measures and a rapid assessment. This continues when the patient is brought into the operating room with staged debridement and reconstruction before extending into the rehabilitation phase. In this phase, functional outcomes and psychosocial adjustments can be identified and addressed. A principles-based approach when dealing with blast injuries to the hand is recommended. These include prioritizing survival and systemic stabilization and defining the zone of injury through physical exam and appropriate imaging. It is important to assess the decision for digit salvage versus long-term function and complication risk. The patient’s psychosocial factors need to be evaluated in addition to having a multidisciplinary team to optimize both physical and psychological recovery.

Role of occupational therapy

OTs as Hand Therapists

Hand therapy is often administered after an injury to the hand to optimize functional outcomes and decrease pain [20]. Hand therapy typically involves several components, including managing edema, caring for the wound, restoring passive and active ROM, performing soft tissue mobilization, providing sensory re-education or desensitization, building strength, and eventually progressing to work-hardening activities [20]. OTs are capable hand therapists, based on the education received, the holistic assessment process, and their unique intervention planning abilities [21]. OTs are especially well-suited for hand rehabilitation because they are trained to address not only the physical components of an injury but also the social and psychological components, as well as how these various components together impact function and engagement in daily occupations [21]. Addressing the psychosocial challenges that accompany hand-related injuries is an essential component of effective hand therapy [15]. For this reason, the ability of an OT to focus on both the physiological and psychosocial processes that occur after hand-related injuries creates especially capable hand therapists.

Rehabilitating the Firework-Injured Hand

The firework-injured hand has been classified as a severe hand injury, which is also referred to as a traumatic hand injury or a mutilating hand injury [22]. By definition, a traumatic hand injury involves extensive damage to multiple hand tissues and leads to significant loss of function and alterations in appearance [18,19]. Traumatic hand injuries often present with extensive skin and soft tissue loss, multiple tendon disruptions with missing tendon segments, nerve damage, fractures or joint dislocations, significant vascular injury leading to devascularized areas, and contamination of the tissues by foreign debris [19]. The rehabilitation process for these types of injuries is complex due to the distinct presentation of each injury; therefore, definitive rehabilitation protocols cannot be established, as each patient requires an individualized treatment plan based on the tissue injury and the tissue healing process [19,22]. However, common factors assessed and addressed include ROM, scar tissue and wound management, activities of daily living, sensation, edema, pain, muscle strength, splinting, and grips, grasps, and pinches [22]. Among the physical factors addressed, there is an identified need to address psychological components as well, due to their influence on the patient’s physical health, well-being, and quality of life [13]. Despite this identified need, psychological concerns are often missed during the evaluative process, resulting in inadequate intervention during treatment [13].

Limitations

This study has several limitations. First, the sample size was relatively small, which may limit the generalizability of our findings to broader populations. Additionally, the cohort was evaluated from a single hospital system and a single surgeon, which could introduce selection bias based on institutional practices and the demographic composition of the patient population. As a result, the findings may not fully reflect the variability in presentation, management, and recovery outcomes seen across more diverse geographic, cultural, and socioeconomic populations. Another limitation was the absence of standardized scoring, such as the Disabilities of the Arm, Shoulder, and Hand (DASH) score for functional recovery and structured assessments for mental health recovery [23]. However, the cases presented add to the understanding of complex hand trauma and highlight the importance of multidisciplinary care.

## Conclusions

Blast-related hand injuries require a structured yet flexible decision-making process that prioritizes survival, carefully defines the zone of injury, and emphasizes overall functional outcomes rather than mere digit salvage. The cases presented demonstrate the importance of early stabilization, staged reconstruction, and realistic prognostic counseling when dealing with such injuries to avoid preventable complications and optimize long-term outcomes. Just as essential is early integration of multidisciplinary care, including occupational hand therapy and psychological support, to address the physical, functional, and psychosocial consequences of these devastating injuries. Due to the varying complexity and severity of such injuries, optimal outcomes are achieved through individualized surgical judgment paired with a coordinated functional and psychological rehabilitation process that maximizes recovery and overall quality of life.
